# The Potential for Connexin Hemichannels to Drive Breast Cancer Progression through Regulation of the Inflammatory Response

**DOI:** 10.3390/ijms19041043

**Published:** 2018-03-30

**Authors:** J. Matthew Rhett, Elizabeth S. Yeh

**Affiliations:** Department of Cell and Molecular Pharmacology & Experimental Therapeutics, Medical University of South Carolina, Charleston, SC 29412, USA; rhettj@musc.edu

**Keywords:** hemichannels, breast cancer, connexin, purinergic, inflammation, tumor-associated macrophage (TAM), tumor-associated neutrophil (TAN), metastasis, resistance

## Abstract

Over the past few decades, connexin hemichannels have become recognized as major players in modulating the inflammatory response. Chronic inflammation is documented to promote tumorigenesis and is a critical component of tumor progression. Furthermore, inflammation is strongly linked to angiogenesis, immunotolerance, invasiveness, metastasis, and resistance in breast cancers. In this review, the literature on the role of connexin hemichannels in inflammation is summarized, and the potential role for hemichannel-mediated inflammation in driving breast cancer progression is discussed. Lastly, the potential for connexin-based therapeutics to modulate the inflammatory component of the tumor microenvironment as an avenue for the treatment of breast cancer is also discussed.

## 1. Introduction

Connexins are membrane proteins that constitute connexin hemichannels and the intercellular channels that comprise gap junction (GJ) structures. These proteins contain a cytoplasmic N-terminus, four transmembrane domains, two extracellular loops that mediate hemichannel docking, a cytoplasmic loop, and a cytoplasmic C-terminus that acts as a regulatory domain [1]. The channels formed from connexins facilitate the passive diffusion of small molecules (<~1000 Da) between the cytoplasm and extracellular space in the case of hemichannels, or between cells in the case of GJ intercellular channels [2]. Much study has been devoted to GJs and their corresponding intercellular channels over the past 70 years, since they were first described in the 1960s. However, the wide-spread study of connexin hemichannels is a much more recent phenomenon, having truly gained traction over the last several decades [3].

Connexin hemichannels have been demonstrated to play both physiological and pathological roles [4]. Physiological functions include the regulation of hearing sensitivity in cochlear cells and ephaptic conduction in the vertebrate retina [5,6]. That said, connexin hemichannels appear to play a key part in mediating a pathological response to injury and stress. Numerous publications have shown that hemichannels open in response to a variety of stimuli that are common features of cell damage including mechanical stimulation, changes in ionic concentration and pH, oxygen and glucose deprivation (i.e., ischemia), and oxidative stress [7,8,9,10]. Hemichannels also open in response to cytokines and inflammatory agents: basic fibroblast growth factor (bFGF), tumor necrosis factor-α, interleukin-1β (IL-1β), and lipopolysaccharide (LPS) [11,12]. The opening of hemichannels results in the diffusion of small molecules such as adenosine triphosphate (ATP) from the cell interior to the extracellular space that can participate in pro-inflammatory and pro-death signaling [13,14].

Oxidative stress and hypoxia are predominant features of the tumor environment, and it is now well established that inflammation plays a role in the initiation and progression of cancer [15,16]. Given the potential connection, it is somewhat surprising that there are only a handful of studies that have examined the role of hemichannels in the development of cancer. In this review, we will discuss the mechanisms by which connexin hemichannels facilitate inflammation, what is currently known about hemichannels and cancer, the role of tumor-associated inflammation in breast cancer, and therapeutics that target hemichannels as adjuvant treatments to minimize tumor-associated inflammation and improve the prognosis for breast cancer patients. We will focus predominantly on the connexin43 (Cx43) isoform as it is the best characterized.

## 2. Connexin Hemichannels and the Inflammatory Response

Connexins have defined expression patterns in the breast. In developed human breast tissue, Cx43 localizes to the myoepithelial cells and fibroblasts and Cx26 localizes to luminal epithelial cells, while Cx30 and Cx32 have been shown to be expressed in luminal epithelial cells during lactation in the mouse [17]. While there is no direct evidence provided in the literature for the presence of hemichannels in these cells, we can likely infer their existence from the presence of functional GJs. Other stromal cells present in the breast that are known to express connexins are resident macrophages and vascular endothelial cells [18,19]. In general, connexin expression appears to be downregulated in primary breast tumors, and the protein that is expressed often appears to be cytoplasmic [20]. These findings initially led to the conclusion that Cx43 was a tumor suppressor; however, a more complex picture has emerged. Both increased and decreased Cx43 expression have been correlated with increased metastatic potential [21]. Given this context, the discrimination of GJ function versus hemichannel function versus channel independent functions may be necessary to create a complete picture of the role of connexins in breast cancer.

The inflammatory response to injured or stressed tissue can generally be characterized by the initial infiltration of the tissue by neutrophils and monocytes (within minutes) followed by macrophages (over a period of days) [22]. If healing progresses normally, the inflammation resolves and damaged tissue is regenerated or replaced with a fibrotic scar (depending on the location and extent of the injury) [23]. A vast array of signaling molecules initiate and coordinate the inflammatory process including bacterial peptides, cytokines, and extracellular nucleic acids (purinergic signaling molecules) [24,25,26]. In particular, it is well documented that connexin hemichannels provide a conduit for extracellular nucleic acids in response to a variety of stimuli. Mechanical stimulation or manipulation of cells has been shown to cause ATP release from corneal endothelial cells, ovarian granulosa cells, astrocytes, and Cx43-expressing C6 glioma cells in a Cx43-dependent manner, and from renal glomerular endothelial cells via Cx40 hemichannels [7,27,28,29]. C6 cells engineered to express Cx43 were demonstrated to release ATP in response to bFGF and LPS in a Cx43-specific manner [11]. Decreased extracellular Ca^2+^ concentration is well documented to induce connexin hemichannel opening and ATP release [3,30,31]. Conversely, increased intracellular calcium induces hemichannel-mediated ATP release [32]. However, increased intracellular Ca^2+^ concentration likely opens hemichannels through a complex biochemical pathway resulting in changes in redox potential, which is another demonstrated hemichannel activator [10,33]. Finally, ischemia-like conditions have been shown to elicit ATP release from connexin hemichannels in cultured cardiomyocytes and astrocytes [34,35,36].

Intracellular ATP provides a primary energy source for cells by acting as an allosteric regulator of enzymes, a substrate in enzymatic reactions, and a building block for nucleic acids. Extracellular ATP’s principal role is as a signaling molecule [37]. Cells express three families of receptors for extracellular nucleotides: P1 adenosine receptors and P2 ATP receptors, which can be broken down in to P2X ion channel receptors, and P2Y G protein coupled receptors (GPCRs) [37]. These receptors have a well-defined physiological role in co-transmission in the central and peripheral nervous system, as well as in the vasculature in regulating vascular tone [38]. In the context of inflammation, extracellular ATP is classified as a damage-associated molecular pattern (DAMP) that alerts host defenses to the presence of injured cells [39]. When cells are ruptured or undergo necrosis, they spill their contents into the extracellular space. This includes cytoplasmic ATP, which can be as high in concentration as 10 mM in intact cells, but its presence outside of cells is tightly regulated by innate mechanisms unless released during cell rupture [40]. This extracellular ATP then acts as a signal for local inflammatory cells, such as resident macrophages, to produce cytokines that in turn attract blood-borne leukocytes, predominantly neutrophils [26]. This source of extracellular ATP is, however, short-lived. Ecto- and exonucleotidases are widely expressed and rapidly convert ATP to adenosine diphosphate (ADP), then adenosine monophosphate (AMP), and finally adenosine [41]. Adenosine is eventually converted to inosine by adenosine deaminase, effectively removing all purines from the signaling pool [42].

In addition to the burst of extracellular ATP produced from ruptured cells, the remaining living cells in the region of tissue surrounding the damage experience many of the cellular stressors discussed above (mechanical stimulation by foreign objects, inflammatory cytokines and bacterial peptides, changes in intra- and extracellular ion concentration, oxidative stress, and lack of oxygen and nutrients) that induce connexin hemichannel-mediated ATP release [13]. This provides a more sustained source of ATP that can directly attract neutrophils and activate macrophages, leading to chronic inflammation if the tissue damage is not resolved [13,25,26,43,44]. Indeed, it has been demonstrated that the inhibition of connexin hemichannels reduces inflammation in a number of injury contexts. The first evidence for connexin hemichannels in inflammation comes from seminal work in which murine dermal wounds were treated with a Cx43 antisense oligonucleotide to locally reduce Cx43 levels, resulting in reduced neutrophil infiltrate in the wound area [45]. However, it should be noted that because the antisense reduced total Cx43 levels, it was not clear whether the effects of the antisense were due to reduced GJ or hemichannel communication, or some combination. Further support for a role for connexin hemichannels in inflammation came from work with the Cx43-mimetic α-connexin carboxyl-terminal peptide 1 (aCT1), which inhibits Cx43 hemichannel function but increases GJ communication [46]. Specifically, it was shown that aCT1 treatment of skin wounds in mice significantly reduced the number of neutrophils in the wound area for up to four days post-injury [47]. Similarly, in a separate study it was found that the application of aCT1 to the site of a silicone implant reduced acute inflammation [48]. Work with another inhibitor of Cx43 hemichannels, juxtamembrane peptide 2 (JM2), demonstrated that inflammation was again reduced and that this reduction was dependent on decreased ATP release from vascular endothelial cells [30,49]. In the context of central nervous system injury, the reduction of total Cx43 with antisense and the blockade of Cx43 hemichannels using mimetic peptides also reduced inflammation [50,51,52]. Importantly, mice with astrocyte-targeted deletion of Cx43 and Cx30 display reduced inflammation following spinal cord injury in association with a significant reduction of ATP release in the injured tissue, as measured by in vivo bioluminescence imaging [53]. 

Many modulators of Cx43 function have moved into clinical trials [54]. Most salient to this review, clinical trials with aCT1 show improved healing of diabetic foot ulcers and venous leg ulcers, which are wounds characterized by chronic inflammatory states [55,56]. Taken together, these data support a model in which early inflammation (i.e., infiltration of neutrophils primarily) is maintained in injured tissue by the secretion of ATP from stressed cells remaining in, and adjacent to, a damaged tissue. Under normal circumstances, as inflammation progresses and converts to the “proliferative” phase of healing, stressors are removed and the early inflammatory response resolves. However, the failure of wound healing to progress leads to chronic inflammation, in part due to continued ATP signaling via connexin hemichannels in stressed cells.

## 3. Inflammation and Breast Cancer

Most cancers are never truly “cured”, but rather “controlled” through cycles of remission and disease recurrence [57,58]. As such, many tumors bear the hallmarks of a chronic wound. Indeed, the stroma surrounding tumors has long been recognized to share similarities with wounded tissue [59]. In particular, leukocytes are well documented to associate with tumors, and have been termed tumor-associated neutrophils and macrophages (TANs and TAMs, respectively) [60,61]. TANs and TAMs may initially be attracted to tumor sites by the presence of DAMPs, including ATP, released by cells in the necrotic core of the tumor, or by tumor cells necrosing in response to surgery, radiation therapy, or chemotherapy [61]. In one example, it was demonstrated that tumor necrosis induced by the inactivation of autophagy in an apoptosis-defective context was associated with macrophage infiltration of the tumor site [62]. Paradoxically, despite the enhanced necrosis, tumor growth was accelerated in this model, suggesting that TAMs promote tumor cell survival and proliferation. In addition, tumors can generate cytokines that attract inflammatory cells including IL-1, IL-6, IL-8, CCL1, CCL2, CCL20, and S100A proteins [60,63,64]. Interestingly, cytokines that attract TANs and TAMs to tumor sites may also be produced by cancer-associated fibroblasts (CAFs) [65,66]. Importantly, Cx43 is expressed in CAFs and may be important for the infiltration of tumor stroma by TAMs [67]. Once TANs and TAMs have arrived at a tumor site, a positive feedback loop may be established in which tumor cells continue to produce signals that attract and maintain inflammatory cells at the site, and TANs and TAMs promote tumor growth by producing growth factors and promoting angiogenesis [15].

TANs and TAMs cells have highly plastic phenotypes, and can play either pro- or anti-tumor progressing roles depending on the type and stage of the cancer involved. TANs and TAMs affect tumor initiation, proliferation, apoptosis, epithelial-to-mesenchymal transition (EMT), angiogenesis, extracellular matrix (ECM) deposition and remodeling, and the ability of adaptive immune cells to respond to the tumor [60,61]. These cells also impact metastasis at the level of intravasation, can chaperone tumor cells through the circulation, and impact the seeding of metastases in the premetastatic niche [68,69,70,71,72]. For the purposes of this review, we will focus on the role of these inflammatory cells as it pertains to breast cancer metastasis and therapeutic resistance. 

Breast cancer is not really a single disease, but a set of cancers that are primarily adenocarcinomas originating from the ductal and lobular tissue of the mammary gland. These cancers can be broken down into molecular subtypes based on their receptor expression pattern: (1) Luminal A tumors tend to express estrogen receptor (ER) and usually progesterone receptor (PR), but are negative for human epidermal growth factor receptor-2 (HER2); (2) Luminal B tumors are usually ER-, PR-, and HER2-positive; (3) HER2 enriched tumors are predominantly positive for HER2, but have little to no ER or PR expression; (4) Basal-like cancers tend to be “triple-negative breast cancers” (TNBCs) that express little to no ER, PR, or HER2 [73]. Inflammatory breast cancer is a rare form of breast cancer that does not fit neatly into the four molecular subtypes. Despite the name, the link between inflammation and inflammatory breast cancer remains poorly understood, but many of the inflammatory pathways that are active in the canonical molecular subtypes also play a role in the tumorigenesis of inflammatory breast cancer [74]. For tumors that are ER-, PR-, and/or HER2-positive, these receptors provide molecular targets for adjuvant treatment, which are designed to block their function [75]. In the case of TNBC, the standard of care remains surgery and chemotherapy [76]. Importantly, receptor positive tumors can become resistant to targeted therapies, and all tumors can recur (often as metastases to other organs or tissues) following a disease-free interval, leading to a chronic disease state [75].

Whether or not a breast tumor develops resistance or metastasizes following treatment is strongly dependent on the presence of TANs and/or TAMs. Generally, the presence of these cells is associated with pro-tumorigenic and metastasis promoting effects. In the case of TANs, studies have shown that a high neutrophil-to-lymphocyte ratio in peripheral blood is prognostic of worse overall survival and disease-free survival for breast cancers, especially ones that are ER- and HER2-negative [77]. Studies using cell-based assays suggest that TANs promote the angiogenesis and invasiveness of breast cancer [78,79]. Further work in mice supports a tumor-promoting role for TANs. One study showed that invasive breast cancer reprogramed myeloid precursors to differentiate into atypical neutrophils (also known as myeloid-derived suppressor cells (MDSCs)) that suppressed the proliferation of CD4- and CD8-positive T-cells by 50%, thus helping tumors to avoid immune detection [80]. Another group found that breast cancers that produced CXCL1 and CXCL2 attracted CD11b^+^Gr1^+^ myeloid cells (neutrophil and monocyte precursors), which in turn promoted tumor cell survival and metastasis [81]. Importantly, chemotherapy induced endothelial cells to produce tumor necrosis factor-α (TNF-α), leading to chemoresistance by “hyperactivating” the system of myeloid cell-stimulated tumor growth and metastasis. 

Similar to the case with neutrophils, TAMs are generally associated with worse outcomes in breast cancer. One study found a significant correlation between TAM infiltration, angiogenic markers, and reduced relapse-free survival and overall survival in clinical breast carcinoma isolates [82]. Direct evidence that TAMs promote breast tumor angiogenesis comes from experiments performed in the mouse mammary tumor virus-polyomavirus middle T-antigen (MMTV-PyMT) mouse model that showed that genetic depletion of macrophages resulted in a significant delay in the development of a dense vascular network and progression to malignancy in primary mammary tumors [83]. Similarly, the depletion of TAMs in murine F9 teratocarcinoma and human A673 rhabdomyosarcoma mouse tumor models resulted in significantly reduced tumor growth and vascularization [84]. Another study also suggested that tumor-associated inflammatory cells can aid metastasis by producing factors that increase vascular permeability [61]. Supporting this statement, direct imaging of vasculature, TAMs, and mammary tumor cells using multiphoton microscopy has shown that cancer cells preferentially intravasate at points in the vasculature where TAMs are located [85].

In addition to promoting angiogenesis and metastasis, inflammatory cells may also contribute to resistance to endocrine therapy. One study showed that ERα-positive, but not ERα-negative, cell lines displayed enhanced growth in response to IL-6 in vitro, and in vivo, MCF-7 cells engineered to express ectopic human IL-6 displayed significantly greater tumor growth in athymic nude mice when compared to parental MCF-7 cells [86]. Later work from the same group demonstrated that IL-6 also promoted EMT in ERα-positive breast cancer cells [87]. Other lines of evidence point to the activation of the transcription factor nuclear factor-κB (NF-κB) in the development of hormone-resistant breast cancer [88]. Importantly, NF-κB can be activated by a number of cytokines produced by TAMs and TANs [61]. TNF-α—another cytokine produced by TAMs—enhances invasiveness of ERα-positive breast cancer cells in vitro [89]. Finally, epidemiological evidence shows that there is a reduced risk of hormone receptor-positive, but not hormone receptor-negative, breast cancer incidence in women who regularly take non-steroidal anti-inflammatory drugs (NSAIDs) [90]. A plausible interpretation of these data is that tumor-associated inflammation, and by extension leukocytes, may provide the signals necessary for hormone-receptor positive mammary tumor cells to evade the targeted chemotherapeutics that make these subtypes of cancer treatable.

It should be noted there is also evidence that tumor-associated inflammatory cells can have anti-metastatic effects. Stockman et al. found that while the deletion of vascular endothelial growth factor-A (VEGF-A) from myeloid cells reduced the vascularization and vascular permeability of mammary tumors in mice, it paradoxically also enhanced tumor growth [91]. This confounding result may be attributed to the authors’ finding that the vasculature of myeloid-derived VEGF-A depleted mice was “normalized” by greater pericyte coverage of the vessels, less leaky vasculature, and reduced hypoxia in the tumors. Neutrophils have also been shown to have anti-metastatic properties. Neutrophil depletion in mice with orthotopically implanted 4T1 tumors displayed a significant reduction in the recruitment of neutrophils to the lungs prior to metastasis at day 7 post-implantation, and significantly increased metastatic load in the lung at day 14, suggesting that “tumor-entrained” neutrophils inhibited the seeding of metastatic cells in the lung [71]. Despite these confounding results, on balance TAMs and TANs appear to promote tumor cell proliferation, tumor-associated angiogenesis, metastasis, and resistance to therapy in breast cancer.

## 4. Hemichannels in Cancer

While the significance of connexins and GJs in cancer has been extensively explored (see [17,21,92,93]), there is limited data from which to draw conclusions about the role of hemichannels in cancer. That said, inferences can be made from a number of sources. For example, antibodies to the extracellular domains of Cx43 have been demonstrated to block hemichannels without affecting gap junctional communication [94]. When Cx43 extracellular loop antibodies (called E2 antibodies) were applied in a rat model of glioblastoma, it was found that animal survival and tumor regression were enhanced, and this effect was synergistic with radiotherapy, suggesting that hemichannel blockade was anti-tumorigenic in this model [95]. However, it should be noted that these results could also be due to an immunogenic response to cells tagged by the antibody. Conversely, additional work has shown that ATP release by hemichannels on osteocytes inhibited the growth, migration, and invasiveness of human breast cancer cells using in vitro cell migration assays [96]. In addition, this study showed that mice with an osteocyte-specific genetic knockout of Cx43, or transgenic mice engineered to have osteocyte-specific expression of a Cx43 mutant (∆130–136) without functional GJ or hemichannels, exhibited enhanced tumor growth of transplanted mammary tumor cells, but not when a GJ-deficient and functional hemichannel Cx43 mutant (R76W) was expressed in osteocytes. These conflicting results suggest that the function of hemichannels in cancer may be specific to the context of a given disease state.

A number of in vitro studies also shed light on the role of hemichannels in cancer. One of the many small molecules that hemichannels can also release is nicotinamide adenine dinucleotide (NAD^+^) [97]. Extracellular NAD^+^ is readily converted to cyclic ADP-ribose (cADPR—a potent second messenger mobilizer of intracellular calcium) by the ectoenzyme CD38, and one study found that NAD^+^ released by CD38^+^ “feeder” cells enhanced the proliferation of CD38^−^ “target” cells, presumably by the conversion of NAD^+^ to cADPR [98]. CD38 is primarily expressed in lymphoid cells (which include T cells and macrophages), and it has been suggested that NAD^+^ released by tumor cells could suppress immune cell function directly while simultaneously enhancing tumor cell proliferation by converting NAD^+^ to cADPR via CD38 on local immune cells [99]. Similarly, ATP that is released by Cx43 hemichannels and converted to adenosine by ectonucleases could provide a pro-metastatic signal in breast cancer by activating purinergic receptors on mammary tumor cells [21]. 

Conversely, it has also been shown that hemichannel opening can provide a pro-death signal. In one instance, localized apoptotic cell death was induced in C6 cells by electroporetic cytochrome C loading. In cells that were engineered to stably express Cx43, cells adjacent to the electroporated cells also underwent apoptosis, but this was not observed in wild-type (non-Cx43-expressing) cells, indicating the spread of some “pro-death” signal through GJs [100]. Interestingly, it was also observed that Cx43-overexpressing cells at distant locations from the cytochrome C loaded cells also underwent apoptosis, and this effect was specifically dependent on Cx43 hemichannel function. The authors of this study hypothesized that a signal was either being released from hemichannels in dying cells of the loading or adjacent region, or being accepted through hemichannels in the distant region. However, further tests to determine the paracrine messenger responsible for transmitting pro-death signals could only rule out ATP and glutamate, suggesting that some other small molecule was responsible or that hemichannel opening itself was contributing to the apoptotic effect. Supporting the second notion, it has also been shown that cultured cardiomyocytes subjected to simulated ischemia-reperfusion display significantly reduced cell viability, and that blocking hemichannels significantly increased cell survival [9]. Taken together, a direct role of connexin hemichannels in cancer has not been well established, but there is strong evidence that hemichannels modulate cellular characteristics that control tumor growth and metastasis.

## 5. The Intersection of Hemichannels, Inflammation, and Breast Cancer: Therapeutic Potential

**Figure 1:**
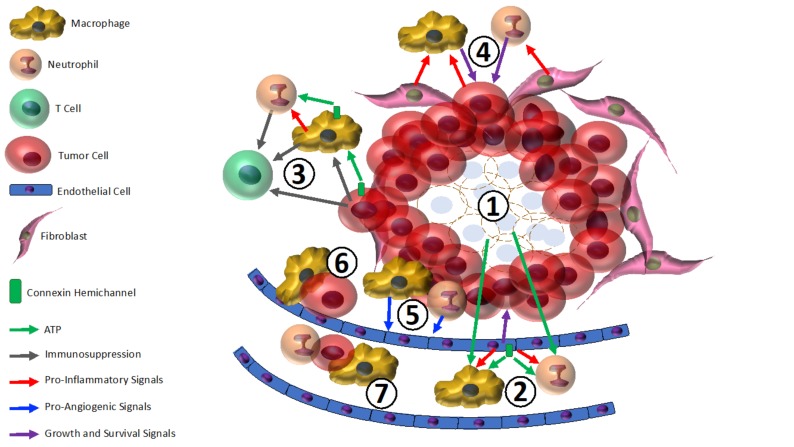
Connexin hemichannels and tumor-associated inflammatory cells in mammary tumor progression. ① Damage-associated molecular patterns (DAMPs), including ATP, that attract and activate macrophages and neutrophils are released from ruptured cells at the necrotic core of the tumor, and from tumor cells necrosing in response to therapy; ② Vascular endothelial cells secrete ATP through hemichannels in response to tumor-related stress, attracting macrophages and neutrophils. Endothelial cells also secrete TNF-α, which promotes tumor growth and inflammation; ③ Tumor cells may release ATP from hemichannels triggered to open by the cellular stressors intrinsic to tumor cell physiology. This can activate local macrophages (i.e., tumor-associated macrophages (TAMs)) that in turn produce pro-inflammatory cytokines. TAMs may also release neutrophil-recruiting ATP from hemichannels. In addition, tumor cells express ectonucleases that convert ATP to adenosine, which has immunosuppressive effects on tumor-associated neutrophils (TANs), TAMs, and T cells; ④ Breast cancer cells and cancer-associated fibroblasts (CAFs) produce pro-inflammatory cytokines that attract and activate TAMs and TANs. In turn, TAMs and TANs produce pro-survival and pro-growth signals that promote tumor progression; ⑤ TAMs and TANs produce pro-angiogenic factors that stimulate tumor vascularization; ⑥ TAMs aid metastatic tumor cells in intravasation; ⑦ TAMs and TANs may chaperone tumor cells through the vasculature to sites of metastasis.

Given that the tumor microenvironment of breast cancer lesions is conducive to the opening of connexin hemichannels [4,16], and these channels are well-established to release pro-inflammatory signals such as ATP [13], it is perhaps unsurprising that malignant breast tumors can be comprised of up to 50% macrophages [101]. Overall, the preponderance of the literature on tumor-associated leukocytes suggests these cells are pro-angiogenic, pro-metastatic, promote tumor growth, and enhance resistance to therapeutics. Therefore, we propose a model in which connexin hemichannels in breast tumors and cells of the surrounding stroma provide a conduit for the secretion of extracellular ATP that attracts TAMs and TANs (Figure 1). In turn, this cascade of events initiated by hemichannels ultimately serves to enhance therapeutic resistance and breast cancer metastasis.

Additionally, it is important to recognize that the breakdown products of ATP also impact mammary tumors. CD39 and CD73 are ectonucleases that convert ATP into ADP, AMP, and adenosine [41]. In particular, adenosine generally has immunosuppressive effects on both innate and adaptive immune cells [102]. With respect to cancer, CD39 and CD73 expression in tumors is correlated with invasiveness and metastasis, and CD73 expression in cultured breast cancer cells enhances invasion, migration, and adhesion [99,103]. Moreover, it was shown in a mouse 4T1.2 breast tumor model that the inhibition of CD73 with CD73-targeted antibodies reduced growth of the primary tumor and impaired metastasis to the lung [104]. These effects were found to occur through the prevention of immunosuppression and the activation of adenosine receptors on the tumor cells.

Although the effects of adenosine are generally considered anti-inflammatory, TAMs exposed to adenosine may actually take on a pro-tumorigenic and pro-metastatic phenotype. Macrophages have been shown to be able to “polarize” into either a pro-inflammatory “M1” phenotype or an alternative “M2” regenerative phenotype, and adenosine promotes the polarization of macrophages into the M2 phenotype [105,106]. Significantly, M2 macrophages stimulate tumor growth, angiogenesis, and are correlated with a poor prognosis in patients [107,108]. Taken together with the preceding discussions, this suggests that the purinergic modulation of leukocyte phenotypes determines whether TAMs and TANs exhibit pro- or anti-tumor characteristics in breast cancer.

As evidenced by the extensive reviews published on the subject, there are a number of connexin-based therapeutics in various phases of development for the treatment of diseases ranging from stroke, to chronic wounds, to cardiovascular disease, and to cancer (see [54,109,110]). Here, the discussion will focus on connexin-based therapeutics that are most likely to have an effect on inflammation in breast cancer. As previously discussed, antibodies against the Cx43 second extracellular loop also inhibit hemichannel function, and are anti-tumorigenic in a rodent glioblastoma model [95]. To our knowledge, there is no direct evidence that this antibody has anti-inflammatory properties, but its ability to inhibit hemichannel activity leaves open the possibility for the E2 antibody to inhibit tumor infiltration and the activation of TAMs and/or TANs, potentially through the disruption of extracellular ATP release [96]. 

The remainder of the discussion will focus on connexin-mimetic peptides due to the non-specific action and limited therapeutic potential of small molecule connexin channel inhibitors (Table 1). As mentioned, aCT1 is a Cx43-mimetic peptide that blocks hemichannels and reduces inflammation in a wound healing context [47,48]. While the effects of aCT1 on inflammation in breast cancer have not been directly explored, prior work has shown that aCT1 enhances the activity of tamoxifen on ER-positive breast cancer cells, and lapatinib on HER2-positive breast cancer cells [111]. These effects were attributed to enhanced GJ coupling. However, since aCT1 also blocks hemichannel activity, it is not possible to conclude the extent to which hemichannel inhibition contributes to the enhanced efficacy of tamoxifen and lapatinib. Similar to the effects on breast cancer cells, aCT1 was shown to induce sensitivity to the front-line glioblastoma therapeutic, temozolomide, in resistant cell lines [112]. Given that aCT1 is anti-inflammatory in other contexts and shows inherent anticancer properties, we speculate that aCT1 could also reduce the leukocyte infiltration of mammary tumors and inhibit tumor progression.

Other connexin-based compounds that target hemichannel function could also potentially reduce TAM and TAN load in breast cancer. Gap junction peptide 26 (Gap26) and Gap27 are mimetics of the Cx43 extracellular loops, and over short time periods specifically inhibit Cx43 hemichannels [113]. In cell culture and organotypic models these peptides can prevent astroglial activation (a marker of neuroinflammation), and Gap27 was found to reduce swelling in a model of spinal cord injury, although it was not further analyzed for its effect on inflammation specifically [36,114,115]. More recently, Gap26 was shown to inhibit the expression of pro-inflammatory cytokines in enteric glia, reduce inflammatory infiltrates in the lungs of mouse models of asthma, and reduce animal mortality in a mouse model of sepsis [116,117,118]. Conversely, the treatment of rat corneal wounds with Gap27 actually increased the accumulation of inflammatory cells [119]. Taken together, these studies largely point to the potential of these peptides to inhibit inflammatory infiltrates in breast cancer, but caution must be taken with these peptides in therapeutic setting as these peptides may have pleiotropic effects on other connexin isoforms [54].

Gap19 is another Cx43-mimetic peptide that, uniquely, contains a sequence from the intracellular loop of Cx43. The initial report on Gap19 was a comprehensive study that showed that Gap19 selectively inhibited Cx43 hemichannels—not GJ communication or other membrane channels—by blocking Cx43 cytoplasmic loop/tail interactions, and decreased infarct size in mouse myocardial ischemia-reperfusion injuries [120]. Subsequent work showed that the peptide also selectively inhibited Cx43 hemichannels in astrocytes [121]. More recently, Gap19 was shown to reduce serum levels of pro-inflammatory cytokines in mouse models of acetaminophen-induced liver injury and non-alcoholic steatohepatitis [122,123]. Conversely, in a mouse model of sepsis, Gap19 was surprisingly found to increase macrophage hemichannel activity and decrease animal survival [118]. Overall, Gap19 shows promise as a therapeutic given its highly selective inhibition of Cx43 hemichannels, but may not be ideal as a means to limit TAM and TAN recruitment and function in tumors due to its hemichannel-activating effect in macrophages.

Another extracellular loop peptide, termed Peptide 5 (P5), has also shown efficacy in reducing inflammation. It was shown to reduce swelling in response to spinal cord injury as well as reduce the number of activated astrocytes in the injury [52,115]. Subsequent work with P5 in stroke models showed neuroprotective effects, but whether this was related to reduced inflammation was not explored [124,125,126]. In a separate study, P5 was shown to inhibit hemichannel-mediated ATP and high mobility group box 1 (HMGB1) release from cultured macrophages, and improved animal survival in models of sepsis and hepatic ischemia-reperfusion injury [118]. Finally, in a rat model of light-induced retinal damage, P5 inhibited inflammatory infiltrates [127]. To our knowledge, no work in cancer has been performed testing this peptide as a therapeutic, but its effectiveness in reducing inflammation points to its potential in modulating the pro-tumorigenic and pro-metastatic effects of TAMs and TANs.

Finally, a peptidomimetic of the Cx43 c-terminal juxtamembrane region (JM2) demonstrated chemotherapeutic potential. As discussed above, JM2 was shown to inhibit hemichannel-mediated ATP release from endothelial cells and reduce inflammation surrounding a submuscular silicone implant in rats [30]. A detailed study of the molecular biology of JM2 revealed that the peptide inhibited the trafficking of Cx43 to the cell surface, thereby inhibiting both Cx43 hemichannel and GJ channel function [49]. Relevant to this discussion, it was found that JM2 impaired Cx43 trafficking by promoting the unrestrained and irrelevant polymerization of microtubules. Indeed, an in vitro assay showed that JM2 enhanced microtubule polymerization to an even greater degree than paclitaxel. These data suggest that, like aCT1, JM2 may inhibit the TAM and TAN infiltration of breast cancer lesions through its inhibitory effect on hemichannel ATP release, but also possesses intrinsic anticancer properties. In summary, a number of promising therapeutic candidates are described for anti-inflammatory properties that have not yet been adopted for testing in cancer. We note that in the case of cancer, in which systemic administration is the preferred delivery route, the rapid degradation of peptides in blood may hamper preclinical studies. However, strategies to protect peptides from proteolysis may improve clinical relevance (e.g., cyclization, L- to D-amino acid replacement, or conjugation to macromolecules) [128].

## 6. Conclusions

The sciences of connexin hemichannels and inflammation in breast cancer have independently grown into their own distinct disciplines over the recent past, but have not yet intersected. Nevertheless, the potential connection is undeniable. Mammary tumors possess the pathophysiological states known to trigger connexin hemichannel opening and ATP release; extracellular ATP is a potent chemoattractant for, and activator of, leukocytes; TAMs and TANs are linked to tumor immunotolerance, angiogenesis, treatment resistance, metastasis, and generally poor prognosis in patients. Future studies should be aimed at dissecting the possible roles of hemichannels in mammary tumor-associated inflammation. Finally, whether connexin-based therapeutics modulate tumor-associated inflammation should be explored. Connexins can be somewhat difficult targets because drugs that affect hemichannels usually also impact GJ communication and vice versa, but hopefully this line of research will produce new chemotherapeutics that reduce the malignancy, resistance, and metastasis of breast cancers.

## Figures and Tables

**Table 1:** Connexin-based therapeutics.

Name	Effects	References	Clinical Trials
Extracellular loop 2 (E2) antibody	Blocks hemichannels without affecting gap junctional (GJ) communication; enhances animal survival and tumor regression in a glioblastoma model; promotes anchorage-independent growth, migration, and invasion of cultured breast cancer cells	[94,95,96]	None
α-connexin carboxyl-terminal peptide 1 (aCT1)	Increases GJ size and gap junctional communication, and concomitantly reduces hemichannel population and cell-extracellular communication; improves wound healing; enhances the activity of targeted therapeutics in breast cancer cells; sensitizes chemoresistant glioblastoma cells to temozolomide	[23,47,48,55,56,111,112]	NCT02652572—Phase I—venus leg ulcers; NCT02652754—Phase I—diabetic foot ulcers; NCT02666131—Phase III—diabetic foot ulcers; NCT02667327—Phase III—diabetic foot ulcers
Gap junction peptide 26 (Gap26)	Blocks hemichannels over short time periods (minutes) and gap junctional communication over long time periods (>30 min); reduces astroglial activation; reduces inflammation; reduces animal cell death due to sepsis	[36,113,116,117,118]	None
Gap junction peptide 27 (Gap27)	Reduces astroglial activation; reduces swelling after spinal cord injury; increases inflammation in corneal wounds	[36,115,119]	None
Gap junction peptide 19 (Gap19)	Selectively inhibits hemichannels and reduces cardiac infarct size; reduces serum levels of pro-inflammatory cytokines due to liver injury; increases macrophage hemichannel activity and decreases animal survival in a rodent sepsis model	[118,120,122,123]	None
Peptide 5 (P5)	Reduces astroglial activation and swelling after spinal cord injury; neuroprotective in stroke models; inhibits hemichannels and improves animal survival in models of sepsis and hepatic ischemia-reperfusion injury; inhibits inflammatory infiltrates into damaged retina	[52,115,118,124,125,126,127]	None
Juxtamembrane peptide 2 (JM2)	Inhibits hemichannels and GJ communication, and promotes microtubule polymerization; inhibits hemichannel-mediated ATP release and reduces inflammation from the foreign body response	[30,49]	None

## References

[1] Giepmans B.N. (2004). Gap junctions and connexin-interacting proteins. Cardiovasc. Res..

[2] Laird D.W. (2006). Life cycle of connexins in health and disease. Biochem. J..

[3] Saez J.C., Leybaert L. (2014). Hunting for connexin hemichannels. FEBS Lett..

[4] Saez J.C., Berthoud V.M., Branes M.C., Martinez A.D., Beyer E.C. (2003). Plasma membrane channels formed by connexins: Their regulation and functions. Physiol. Rev..

[5] Anselmi F., Hernandez V.H., Crispino G., Seydel A., Ortolano S., Roper S.D., Kessaris N., Richardson W., Rickheit G., Filippov M.A. (2008). ATP release through connexin hemichannels and gap junction transfer of second messengers propagate Ca^2+^ signals across the inner ear. Proc. Natl. Acad. Sci. USA.

[6] Klaassen L.J., Sun Z., Steijaert M.N., Bolte P., Fahrenfort I., Sjoerdsma T., Klooster J., Claassen Y., Shields C.R., Ten Eikelder H.M. (2011). Synaptic transmission from horizontal cells to cones is impaired by loss of connexin hemichannels. PLoS Biol..

[7] Gomes P., Srinivas S.P., Van Driessche W., Vereecke J., Himpens B. (2005). ATP release through connexin hemichannels in corneal endothelial cells. Investig. Ophthalmol. Vis. Sci..

[8] Li H., Liu T.F., Lazrak A., Peracchia C., Goldberg G.S., Lampe P.D., Johnson R.G. (1996). Properties and regulation of gap junctional hemichannels in the plasma membranes of cultured cells. J. Cell Biol..

[9] Shintani-Ishida K., Uemura K., Yoshida K. (2007). Hemichannels in cardiomyocytes open transiently during ischemia and contribute to reperfusion injury following brief ischemia. Am. J. Physiol. Heart Circ. Physiol..

[10] Retamal M.A., Schalper K.A., Shoji K.F., Bennett M.V., Saez J.C. (2007). Opening of connexin 43 hemichannels is increased by lowering intracellular redox potential. Proc. Natl. Acad. Sci. USA.

[11] De Vuyst E., Decrock E., de Bock M., Yamasaki H., Naus C.C., Evans W.H., Leybaert L. (2007). Connexin hemichannels and gap junction channels are differentially influenced by lipopolysaccharide and basic fibroblast growth factor. Mol. Biol. Cell.

[12] Retamal M.A., Froger N., Palacios-Prado N., Ezan P., Saez P.J., Saez J.C., Giaume C. (2007). Cx43 hemichannels and gap junction channels in astrocytes are regulated oppositely by proinflammatory cytokines released from activated microglia. J. Neurosci..

[13] Rhett J.M., Fann S.A., Yost M.J. (2014). Purinergic signaling in early inflammatory events of the foreign body response: Modulating extracellular ATP as an enabling technology for engineered implants and tissues. Tissue Eng. Part B Rev..

[14] Cotrina M.L., Lin J.H., Nedergaard M. (2008). Adhesive properties of connexin hemichannels. Glia.

[15] Taniguchi K., Karin M. (2018). NF-κB, inflammation, immunity and cancer: Coming of age. Nat. Rev. Immunol..

[16] Grek C.L., Tew K.D. (2010). Redox metabolism and malignancy. Curr. Opin. Pharmacol..

[17] McLachlan E., Shao Q., Laird D.W. (2007). Connexins and gap junctions in mammary gland development and breast cancer progression. J. Membr. Biol..

[18] Westphalen K., Gusarova G.A., Islam M.N., Subramanian M., Cohen T.S., Prince A.S., Bhattacharya J. (2014). Sessile alveolar macrophages communicate with alveolar epithelium to modulate immunity. Nature.

[19] Lohman A.W., Billaud M., Isakson B.E. (2012). Mechanisms of ATP release and signalling in the blood vessel wall. Cardiovasc. Res..

[20] Phillips S.L., Williams C.B., Zambrano J.N., Williams C.J., Yeh E.S. (2017). Connexin 43 in the development and progression of breast cancer: What’s the connection? (Review). Int. J. Oncol..

[21] Grek C.L., Rhett J.M., Bruce J.S., Ghatnekar G.S., Yeh E.S. (2016). Connexin 43, breast cancer tumor suppressor: Missed connections?. Cancer Lett..

[22] Clark R., Clark R. (1996). Wound Repair Overview and General Considerations. The Molecular and Cellular Biology of Wound Repair.

[23] Rhett J.M., Ghatnekar G.S., Palatinus J.A., O’Quinn M., Yost M.J., Gourdie R.G. (2008). Novel therapies for scar reduction and regenerative healing of skin wounds. Trends Biotechnol..

[24] Newton K., Dixit V.M. (2012). Signaling in innate immunity and inflammation. Cold Spring Harb. Perspect. Biol..

[25] Eltzschig H.K., Sitkovsky M.V., Robson S.C. (2012). Purinergic signaling during inflammation. N. Engl. J. Med..

[26] McDonald B., Pittman K., Menezes G.B., Hirota S.A., Slaba I., Waterhouse C.C., Beck P.L., Muruve D.A., Kubes P. (2010). Intravascular danger signals guide neutrophils to sites of sterile inflammation. Science.

[27] Tong D., Li T.Y., Naus K.E., Bai D., Kidder G.M. (2007). In vivo analysis of undocked connexin43 gap junction hemichannels in ovarian granulosa cells. J. Cell Sci..

[28] Stout C.E., Costantin J.L., Naus C.C., Charles A.C. (2002). Intercellular calcium signaling in astrocytes via ATP release through connexin hemichannels. J. Biol. Chem..

[29] Toma I., Bansal E., Meer E.J., Kang J.J., Vargas S.L., Peti-Peterdi J. (2008). Connexin 40 and ATP-dependent intercellular calcium wave in renal glomerular endothelial cells. Am. J. Physiol. Regul. Integr. Comp. Physiol..

[30] Calder B.W., Matthew Rhett J., Bainbridge H., Fann S.A., Gourdie R.G., Yost M.J. (2015). Inhibition of connexin 43 hemichannel-mediated ATP release attenuates early inflammation during the foreign body response. Tissue Eng. Part A.

[31] Saez J.C., Retamal M.A., Basilio D., Bukauskas F.F., Bennett M.V. (2005). Connexin-based gap junction hemichannels: Gating mechanisms. Biochim. Biophys. Acta.

[32] Godecke S., Roderigo C., Rose C.R., Rauch B.H., Godecke A., Schrader J. (2012). Thrombin-induced ATP release from human umbilical vein endothelial cells. Am. J. Physiol. Cell Physiol..

[33] De Vuyst E., Wang N., Decrock E., de Bock M., Vinken M., van Moorhem M., Lai C., Culot M., Rogiers V., Cecchelli R. (2009). Ca^2+^ regulation of connexin 43 hemichannels in C6 glioma and glial cells. Cell Calcium.

[34] Clarke T.C., Williams O.J., Martin P.E., Evans W.H. (2009). ATP release by cardiac myocytes in a simulated ischaemia model: Inhibition by a connexin mimetic and enhancement by an antiarrhythmic peptide. Eur. J. Pharmacol..

[35] Contreras J.E., Sanchez H.A., Eugenin E.A., Speidel D., Theis M., Willecke K., Bukauskas F.F., Bennett M.V., Saez J.C. (2002). Metabolic inhibition induces opening of unapposed connexin 43 gap junction hemichannels and reduces gap junctional communication in cortical astrocytes in culture. Proc. Natl. Acad. Sci. USA.

[36] Orellana J.A., Froger N., Ezan P., Jiang J.X., Bennett M.V., Naus C.C., Giaume C., Saez J.C. (2011). ATP and glutamate released via astroglial connexin 43 hemichannels mediate neuronal death through activation of pannexin 1 hemichannels. J. Neurochem..

[37] Burnstock G. (2012). Purinergic signalling: Its unpopular beginning, its acceptance and its exciting future. Bioessays.

[38] Burnstock G. (2011). Introductory overview of purinergic signalling. Front. Biosci. (Elite Ed.).

[39] Chen G.Y., Nunez G. (2010). Sterile inflammation: Sensing and reacting to damage. Nat. Rev. Immunol..

[40] Beis I., Newsholme E.A. (1975). The contents of adenine nucleotides, phosphagens and some glycolytic intermediates in resting muscles from vertebrates and invertebrates. Biochem. J..

[41] Regateiro F.S., Cobbold S.P., Waldmann H. (2013). CD73 and adenosine generation in the creation of regulatory microenvironments. Clin. Exp. Immunol..

[42] Moser G.H., Schrader J., Deussen A. (1989). Turnover of adenosine in plasma of human and dog blood. Am. J. Physiol..

[43] Chen Y., Corriden R., Inoue Y., Yip L., Hashiguchi N., Zinkernagel A., Nizet V., Insel P.A., Junger W.G. (2006). ATP release guides neutrophil chemotaxis via P2Y2 and A3 receptors. Science.

[44] Chen Y., Yao Y., Sumi Y., Li A., To U.K., Elkhal A., Inoue Y., Woehrle T., Zhang Q., Hauser C. (2010). Purinergic signaling: A fundamental mechanism in neutrophil activation. Sci. Signal..

[45] Qiu C., Coutinho P., Frank S., Franke S., Law L.Y., Martin P., Green C.R., Becker D.L. (2003). Targeting connexin43 expression accelerates the rate of wound repair. Curr. Biol..

[46] Rhett J.M., Jourdan J., Gourdie R.G. (2011). Connexin 43 connexon to gap junction transition is regulated by zonula occludens-1. Mol. Biol. Cell.

[47] Ghatnekar G.S., O’Quinn M.P., Jourdan L.J., Gurjarpadhye A.A., Draughn R.L., Gourdie R.G. (2009). Connexin43 carboxyl-terminal peptides reduce scar progenitor and promote regenerative healing following skin wounding. Regen. Med..

[48] Soder B.L., Propst J.T., Brooks T.M., Goodwin R.L., Friedman H.I., Yost M.J., Gourdie R.G. (2009). The connexin43 carboxyl-terminal peptide ACT1 modulates the biological response to silicone implants. Plast. Reconstr. Surg..

[49] Rhett J.M., Calder B.W., Fann S.A., Bainbridge H., Gourdie R.G., Yost M.J. (2017). Mechanism of action of the anti-inflammatory connexin43 mimetic peptide JM2. Am. J. Physiol. Cell Physiol..

[50] Cronin M., Anderson P.N., Cook J.E., Green C.R., Becker D.L. (2008). Blocking connexin43 expression reduces inflammation and improves functional recovery after spinal cord injury. Mol. Cell. Neurosci..

[51] Danesh-Meyer H.V., Kerr N.M., Zhang J., Eady E.K., O’Carroll S.J., Nicholson L.F., Johnson C.S., Green C.R. (2012). Connexin43 mimetic peptide reduces vascular leak and retinal ganglion cell death following retinal ischaemia. Brain.

[52] Mao Y., Tonkin R.S., Nguyen T., O’Carroll S.J., Nicholson L.F., Green C.R., Moalem-Taylor G., Gorrie C.A. (2017). Systemic Administration of Connexin43 Mimetic Peptide Improves Functional Recovery after Traumatic Spinal Cord Injury in Adult Rats. J. Neurotrauma.

[53] Huang C., Han X., Li X., Lam E., Peng W., Lou N., Torres A., Yang M., Garre J.M., Tian G.F. (2012). Critical role of connexin 43 in secondary expansion of traumatic spinal cord injury. J. Neurosci..

[54] Grek C.L., Rhett J.M., Ghatnekar G.S. (2014). Cardiac to cancer: Connecting connexins to clinical opportunity. FEBS Lett..

[55] Grek C.L., Prasad G.M., Viswanathan V., Armstrong D.G., Gourdie R.G., Ghatnekar G.S. (2015). Topical administration of a connexin43-based peptide augments healing of chronic neuropathic diabetic foot ulcers: A multicenter, randomized trial. Wound Repair Regen..

[56] Ghatnekar G.S., Grek C.L., Armstrong D.G., Desai S.C., Gourdie R.G. (2015). The effect of a connexin43-based Peptide on the healing of chronic venous leg ulcers: A multicenter, randomized trial. J. Investig. Dermatol..

[57] Managing Cancer as a Chronic Illness. https://www.cancer.org/treatment/survivorship-during-and-after-treatment/when-cancer-doesnt-go-away.html.

[58] Cheng Y.C., Ueno N.T. (2012). Improvement of survival and prospect of cure in patients with metastatic breast cancer. Breast Cancer.

[59] Dvorak H.F. (2015). Tumors: Wounds that do not heal-redux. Cancer Immunol. Res..

[60] Galdiero M.R., Varricchi G., Loffredo S., Mantovani A., Marone G. (2018). Roles of neutrophils in cancer growth and progression. J. Leukoc. Biol..

[61] Grivennikov S.I., Greten F.R., Karin M. (2010). Immunity, inflammation, and cancer. Cell.

[62] Degenhardt K., Mathew R., Beaudoin B., Bray K., Anderson D., Chen G., Mukherjee C., Shi Y., Gelinas C., Fan Y. (2006). Autophagy promotes tumor cell survival and restricts necrosis, inflammation, and tumorigenesis. Cancer Cell.

[63] Mantovani A., Allavena P., Sica A., Balkwill F. (2008). Cancer-related inflammation. Nature.

[64] Bonecchi R. (2009). Chemokines and chemokine receptors: An overview. Front. Biosci..

[65] Liao D., Luo Y., Markowitz D., Xiang R., Reisfeld R.A. (2009). Cancer associated fibroblasts promote tumor growth and metastasis by modulating the tumor immune microenvironment in a 4T1 murine breast cancer model. PLoS ONE.

[66] Orimo A., Gupta P.B., Sgroi D.C., Arenzana-Seisdedos F., Delaunay T., Naeem R., Carey V.J., Richardson A.L., Weinberg R.A. (2005). Stromal fibroblasts present in invasive human breast carcinomas promote tumor growth and angiogenesis through elevated SDF-1/CXCL12 secretion. Cell.

[67] Essa A.A., Yamazaki M., Maruyama S., Abe T., Babkair H., Raghib A.M., Megahed E.M., Cheng J., Saku T. (2016). Tumour-associated macrophages are recruited and differentiated in the neoplastic stroma of oral squamous cell carcinoma. Pathology.

[68] Bekes E.M., Schweighofer B., Kupriyanova T.A., Zajac E., Ardi V.C., Quigley J.P., Deryugina E.I. (2011). Tumor-recruited neutrophils and neutrophil TIMP-free MMP-9 regulate coordinately the levels of tumor angiogenesis and efficiency of malignant cell intravasation. Am. J. Pathol..

[69] Condeelis J., Pollard J.W. (2006). Macrophages: Obligate partners for tumor cell migration, invasion, and metastasis. Cell.

[70] Cools-Lartigue J., Spicer J., McDonald B., Gowing S., Chow S., Giannias B., Bourdeau F., Kubes P., Ferri L. (2013). Neutrophil extracellular traps sequester circulating tumor cells and promote metastasis. J. Clin. Investig..

[71] Granot Z., Henke E., Comen E.A., King T.A., Norton L., Benezra R. (2011). Tumor entrained neutrophils inhibit seeding in the premetastatic lung. Cancer Cell.

[72] Kaplan R.N., Riba R.D., Zacharoulis S., Bramley A.H., Vincent L., Costa C., MacDonald D.D., Jin D.K., Shido K., Kerns S.A. (2005). VEGFR1-positive haematopoietic bone marrow progenitors initiate the pre-metastatic niche. Nature.

[73] Dai X., Xiang L., Li T., Bai Z. (2016). Cancer Hallmarks, Biomarkers and Breast Cancer Molecular Subtypes. J. Cancer.

[74] Fouad T.M., Kogawa T., Reuben J.M., Ueno N.T. (2014). The role of inflammation in inflammatory breast cancer. Adv. Exp. Med. Biol..

[75] Apuri S. (2017). Neoadjuvant and Adjuvant Therapies for Breast Cancer. South. Med. J..

[76] Wahba H.A., El-Hadaad H.A. (2015). Current approaches in treatment of triple-negative breast cancer. Cancer Biol. Med..

[77] Ethier J.L., Desautels D., Templeton A., Shah P.S., Amir E. (2017). Prognostic role of neutrophil-to-lymphocyte ratio in breast cancer: A systematic review and meta-analysis. Breast Cancer Res..

[78] Ardi V.C., Kupriyanova T.A., Deryugina E.I., Quigley J.P. (2007). Human neutrophils uniquely release TIMP-free MMP-9 to provide a potent catalytic stimulator of angiogenesis. Proc. Natl. Acad. Sci. USA.

[79] Queen M.M., Ryan R.E., Holzer R.G., Keller-Peck C.R., Jorcyk C.L. (2005). Breast cancer cells stimulate neutrophils to produce oncostatin M: Potential implications for tumor progression. Cancer Res..

[80] Casbon A.J., Reynaud D., Park C., Khuc E., Gan D.D., Schepers K., Passegue E., Werb Z. (2015). Invasive breast cancer reprograms early myeloid differentiation in the bone marrow to generate immunosuppressive neutrophils. Proc. Natl. Acad. Sci. USA.

[81] Acharyya S., Oskarsson T., Vanharanta S., Malladi S., Kim J., Morris P.G., Manova-Todorova K., Leversha M., Hogg N., Seshan V.E. (2012). A CXCL1 paracrine network links cancer chemoresistance and metastasis. Cell.

[82] Leek R.D., Lewis C.E., Whitehouse R., Greenall M., Clarke J., Harris A.L. (1996). Association of macrophage infiltration with angiogenesis and prognosis in invasive breast carcinoma. Cancer Res..

[83] Lin E.Y., Li J.F., Gnatovskiy L., Deng Y., Zhu L., Grzesik D.A., Qian H., Xue X.N., Pollard J.W. (2006). Macrophages regulate the angiogenic switch in a mouse model of breast cancer. Cancer Res..

[84] Zeisberger S.M., Odermatt B., Marty C., Zehnder-Fjallman A.H., Ballmer-Hofer K., Schwendener R.A. (2006). Clodronate-liposome-mediated depletion of tumour-associated macrophages: A new and highly effective antiangiogenic therapy approach. Br. J. Cancer.

[85] Wyckoff J.B., Wang Y., Lin E.Y., Li J.F., Goswami S., Stanley E.R., Segall J.E., Pollard J.W., Condeelis J. (2007). Direct visualization of macrophage-assisted tumor cell intravasation in mammary tumors. Cancer Res..

[86] Sasser A.K., Sullivan N.J., Studebaker A.W., Hendey L.F., Axel A.E., Hall B.M. (2007). Interleukin-6 is a potent growth factor for ER-α-positive human breast cancer. FASEB J..

[87] Sullivan N.J., Sasser A.K., Axel A.E., Vesuna F., Raman V., Ramirez N., Oberyszyn T.M., Hall B.M. (2009). Interleukin-6 induces an epithelial-mesenchymal transition phenotype in human breast cancer cells. Oncogene.

[88] Zhou Y., Eppenberger-Castori S., Eppenberger U., Benz C.C. (2005). The NF-κB pathway and endocrine-resistant breast cancer. Endocr. Relat. Cancer.

[89] Yin Y., Chen X., Shu Y. (2009). Gene expression of the invasive phenotype of TNF-α-treated MCF-7 cells. Biomed. Pharmacother..

[90] Terry M.B., Gammon M.D., Zhang F.F., Tawfik H., Teitelbaum S.L., Britton J.A., Subbaramaiah K., Dannenberg A.J., Neugut A.I. (2004). Association of frequency and duration of aspirin use and hormone receptor status with breast cancer risk. JAMA.

[91] Stockmann C., Doedens A., Weidemann A., Zhang N., Takeda N., Greenberg J.I., Cheresh D.A., Johnson R.S. (2008). Deletion of vascular endothelial growth factor in myeloid cells accelerates tumorigenesis. Nature.

[92] Naus C.C., Laird D.W. (2010). Implications and challenges of connexin connections to cancer. Nat. Rev. Cancer.

[93] Jiang J.X., Penuela S. (2016). Connexin and pannexin channels in cancer. BMC Cell Biol..

[94] Siller-Jackson A.J., Burra S., Gu S., Xia X., Bonewald L.F., Sprague E., Jiang J.X. (2008). Adaptation of connexin 43-hemichannel prostaglandin release to mechanical loading. J. Biol. Chem..

[95] Yusubalieva G.M., Baklaushev V.P., Gurina O.I., Zorkina Y.A., Gubskii I.L., Kobyakov G.L., Golanov A.V., Goryainov S.A., Gorlachev G.E., Konovalov A.N. (2014). Treatment of poorly differentiated glioma using a combination of monoclonal antibodies to extracellular connexin-43 fragment, temozolomide, and radiotherapy. Bull. Exp. Biol. Med..

[96] Zhou J.Z., Riquelme M.A., Gu S., Kar R., Gao X., Sun L., Jiang J.X. (2016). Osteocytic connexin hemichannels suppress breast cancer growth and bone metastasis. Oncogene.

[97] Bruzzone S., Guida L., Zocchi E., Franco L., De Flora A. (2001). Connexin 43 hemi channels mediate Ca^2+^-regulated transmembrane NAD^+^ fluxes in intact cells. FASEB J..

[98] Franco L., Zocchi E., Usai C., Guida L., Bruzzone S., Costa A., De Flora A. (2001). Paracrine roles of NAD^+^ and cyclic ADP-ribose in increasing intracellular calcium and enhancing cell proliferation of 3T3 fibroblasts. J. Biol. Chem..

[99] Schalper K.A., Carvajal-Hausdorf D., Oyarzo M.P. (2014). Possible role of hemichannels in cancer. Front. Physiol..

[100] Decrock E., De Vuyst E., Vinken M., Van Moorhem M., Vranckx K., Wang N., Van Laeken L., De Bock M., D’Herde K., Lai C.P. (2009). Connexin 43 hemichannels contribute to the propagation of apoptotic cell death in a rat C6 glioma cell model. Cell Death Differ..

[101] Kelly P.M., Davison R.S., Bliss E., McGee J.O. (1988). Macrophages in human breast disease: A quantitative immunohistochemical study. Br. J. Cancer.

[102] Junger W.G. (2011). Immune cell regulation by autocrine purinergic signalling. Nat. Rev. Immunol..

[103] Wang L., Zhou X., Zhou T., Ma D., Chen S., Zhi X., Yin L., Shao Z., Ou Z., Zhou P. (2008). Ecto-5′-nucleotidase promotes invasion, migration and adhesion of human breast cancer cells. J. Cancer Res. Clin. Oncol..

[104] Stagg J., Divisekera U., McLaughlin N., Sharkey J., Pommey S., Denoyer D., Dwyer K.M., Smyth M.J. (2010). Anti-CD73 antibody therapy inhibits breast tumor growth and metastasis. Proc. Natl. Acad. Sci. USA.

[105] Brown B.N., Ratner B.D., Goodman S.B., Amar S., Badylak S.F. (2012). Macrophage polarization: An opportunity for improved outcomes in biomaterials and regenerative medicine. Biomaterials.

[106] Csoka B., Selmeczy Z., Koscso B., Nemeth Z.H., Pacher P., Murray P.J., Kepka-Lenhart D., Morris S.M., Gause W.C., Leibovich S.J. (2012). Adenosine promotes alternative macrophage activation via A2A and A2B receptors. FASEB J..

[107] Choi J., Gyamfi J., Jang H., Koo J.S. (2018). The role of tumor-associated macrophage in breast cancer biology. Histol. Histopathol..

[108] Komohara Y., Jinushi M., Takeya M. (2014). Clinical significance of macrophage heterogeneity in human malignant tumors. Cancer Sci..

[109] Evans W.H., Bultynck G., Leybaert L. (2012). Manipulating connexin communication channels: Use of peptidomimetics and the translational outputs. J. Membr. Biol..

[110] De Vuyst E., Boengler K., Antoons G., Sipido K.R., Schulz R., Leybaert L. (2011). Pharmacological modulation of connexin-formed channels in cardiac pathophysiology. Br. J. Pharmacol..

[111] Grek C.L., Rhett J.M., Bruce J.S., Abt M.A., Ghatnekar G.S., Yeh E.S. (2015). Targeting connexin 43 with α-connexin carboxyl-terminal (ACT1) peptide enhances the activity of the targeted inhibitors, tamoxifen and lapatinib, in breast cancer: Clinical implication for ACT1. BMC Cancer.

[112] Murphy S.F., Varghese R.T., Lamouille S., Guo S., Pridham K.J., Kanabur P., Osimani A.M., Sharma S., Jourdan J., Rodgers C.M. (2016). Connexin 43 Inhibition Sensitizes Chemoresistant Glioblastoma Cells to Temozolomide. Cancer Res..

[113] Desplantez T., Verma V., Leybaert L., Evans W.H., Weingart R. (2012). Gap26, a connexin mimetic peptide, inhibits currents carried by connexin43 hemichannels and gap junction channels. Pharmacol. Res..

[114] Orellana J.A., Shoji K.F., Abudara V., Ezan P., Amigou E., Saez P.J., Jiang J.X., Naus C.C., Saez J.C., Giaume C. (2011). Amyloid β-induced death in neurons involves glial and neuronal hemichannels. J. Neurosci..

[115] O’Carroll S.J., Alkadhi M., Nicholson L.F., Green C.R. (2008). Connexin 43 mimetic peptides reduce swelling, astrogliosis, and neuronal cell death after spinal cord injury. Cell Commun. Adhes..

[116] Bhave S., Gade A., Kang M., Hauser K.F., Dewey W.L., Akbarali H.I. (2017). Connexin-purinergic signaling in enteric glia mediates the prolonged effect of morphine on constipation. FASEB J..

[117] Huang J.-Q., Chen X.-Y., Huang F., Fan J.-M., Shi X.-W., Ju Y.-K. (2018). Effects of Connexin 43 Inhibition in an Ovalbumin-induced Mouse Model of Asthma. Iranian J. Allergy Asthma Immunol..

[118] Li W., Bao G., Chen W., Qiang X., Zhu S., Wang S., He M., Ma G., Ochani M., Al-Abed Y. (2018). Connexin 43 Hemichannel as a Novel Mediator of Sterile and Infectious Inflammatory Diseases. Sci. Rep..

[119] Elbadawy H.M., Mirabelli P., Xeroudaki M., Parekh M., Bertolin M., Breda C., Cagini C., Ponzin D., Lagali N., Ferrari S. (2016). Effect of connexin 43 inhibition by the mimetic peptide Gap27 on corneal wound healing, inflammation and neovascularization. Br. J. Pharmacol..

[120] Wang N., de Vuyst E., Ponsaerts R., Boengler K., Palacios-Prado N., Wauman J., Lai C.P., de Bock M., Decrock E., Bol M. (2013). Selective inhibition of Cx43 hemichannels by Gap19 and its impact on myocardial ischemia/reperfusion injury. Basic Res. Cardiol..

[121] Abudara V., Bechberger J., Freitas-Andrade M., de Bock M., Wang N., Bultynck G., Naus C.C., Leybaert L., Giaume C. (2014). The connexin43 mimetic peptide Gap19 inhibits hemichannels without altering gap junctional communication in astrocytes. Front. Cell. Neurosci..

[122] Maes M., Crespo Yanguas S., Willebrords J., Weemhoff J.L., da Silva T.C., Decrock E., Lebofsky M., Pereira I.V.A., Leybaert L., Farhood A. (2017). Connexin hemichannel inhibition reduces acetaminophen-induced liver injury in mice. Toxicol. Lett..

[123] Willebrords J., Cogliati B., Pereira I.V.A., da Silva T.C., Crespo Yanguas S., Maes M., Govoni V.M., Lima A., Felisbino D.A., Decrock E. (2017). Inhibition of connexin hemichannels alleviates non-alcoholic steatohepatitis in mice. Sci. Rep..

[124] Davidson J.O., Green C.R., Nicholson L.F., Bennet L., Gunn A.J. (2012). Deleterious effects of high dose connexin 43 mimetic Peptide infusion after cerebral ischaemia in near-term fetal sheep. Int. J. Mol. Sci..

[125] Davidson J.O., Green C.R., Nicholson L.F., Bennet L., Gunn A.J. (2013). Connexin hemichannel blockade is neuroprotective after, but not during, global cerebral ischemia in near-term fetal sheep. Exp. Neurol..

[126] Davidson J.O., Green C.R., Nicholson L.F., O’Carroll S.J., Fraser M., Bennet L., Gunn A.J. (2012). Connexin hemichannel blockade improves outcomes in a model of fetal ischemia. Ann. Neurol..

[127] Guo C.X., Mat Nor M.N., Danesh-Meyer H.V., Vessey K.A., Fletcher E.L., O’Carroll S.J., Acosta M.L., Green C.R. (2016). Connexin43 Mimetic Peptide Improves Retinal Function and Reduces Inflammation in a Light-Damaged Albino Rat Model. Investig. Ophthalmol. Vis. Sci..

[128] Di L. (2015). Strategic approaches to optimizing peptide ADME properties. AAPS J..

